# Developing and validating a SNARE-based prognostic model to forecast outcomes and immune microenvironment in lung squamous cell carcinoma

**DOI:** 10.1097/MD.0000000000049394

**Published:** 2026-07-03

**Authors:** Qingqing Chen, Jian Wang, Xiaoting Wu

**Affiliations:** a Department of Respiratory and Critical Care Medicine, The First People’s Hospital of Linping District, Hangzhou, Zhejiang, China.

**Keywords:** immune infiltration, LASSO, lung squamous cell carcinoma, nomogram, SNARE

## Abstract

SNARE-related genes (SRGs), which mediate membrane fusion events, play critical roles in the development and advancement of cancer. The aim of this research was to develop a prognostic signature based on SRGs for lung squamous cell carcinoma (LUSC) and assess the biological functions of associated SRGs. Transcriptomic and clinical data for LUSC were obtained from public databases. Least absolute shrinkage and selection operator regression generated a risk signature, which was analyzed for associations with clinicopathological features, immune microenvironment, and drug sensitivity. A nomogram was developed based on SRGs-derived signature. Biological function analysis was performed using RNA interference techniques, colony formation assays, transwell assays, and wound healing assays. Ten significantly prognostic SRGs were identified and employed to generate a risk score for LUSC. This signature categorized LUSC patients into the high- and low-risk cohorts, demonstrating notable variations in clinical-pathological features, immune infiltration patterns, gene expression profiles, and responses to drug sensitivity. The developed signature proved itself as an independent predictor of LUSC prognosis, exhibiting robust predictive power for survival outcomes. A nomogram model combining the signature with smoking history showed superior performance and clinical applicability. Knockdown of the bet1 golgi vesicular membrane trafficking protein (*BET1*) gene in LUSC cells significantly enhanced clonogenic capacity and promoted cancer cell migration and invasion. Our study substantiated the potential clinical significance of a risk signature derived from SRGs in LUSC’ prognosis prediction. It provided novel insights into the interactions among SRGs, tumors, and immunity, potentially contributing to the advancement of precision medicine for LUSC.

## 1. Introduction

Lung cancer ranks first in mortality rates and second in incidence among malignant tumors globally.^[1]^ Non-small cell lung cancer (NSCLC) constitutes over 85% of cases, with lung squamous cell carcinoma (LUSC) representing 30% of NSCLC subtypes.^[2]^ Most LUSC patients are diagnosed at advanced stages, where conventional therapies show limited efficacy.^[3]^ Although immunotherapy improves survival,^[4]^ advanced LUSC may resist programmed cell death protein 1 (PD-1) or programmed death-ligand 1 (PD-L1) checkpoint blockade.^[5]^ Consequently, identifying new biomarkers for early detection and therapeutic targets in LUSC is of paramount importance.

Vesicular trafficking is essential for normal cellular activities, encompassing growth, division, motility, and intercellular communication.^[6]^ Soluble N-ethylmaleimide-sensitive factor attachment protein receptors (SNAREs) are key members of a protein family critical for mediating membrane fusion events during vesicular transport.^[7]^ Reports have shown that SNARE-related genes (SRGs) play roles in various cancers, including colorectal, breast, and pancreatic cancer.^[8–10]^ However, the specific functions and prognostic values of SRGs in LUSC remain elusive.

The principal focus of this study lies in assessing the prognostic significance of SRGs in LUSC patients via comprehensive bioinformatics analysis and experimental verification. We aim to construct a risk signature derived from SRGs and explore its correlations with clinical-pathological features, the tumor microenvironment, and drug responses. Furthermore, we validated the biological functions of SRGs in the signature.

## 2. Materials and methods

### 2.1. Datasets and processing

Gene expression data, clinical details, and survival records for LUSC patients were acquired from the University of California Santa Cruz Xena database (https://xenabrowser.net/datapages/) as the primary training set. Subsequently, these TCGA-LUSC data underwent log2-transformed for downstream analysis. For validation purposes, the GSE73403 and GSE30219 datasets were sourced from the Gene Expression Omnibus (https://www.ncbi.nlm.nih.gov/gds/) database. To identify SRGs, we accessed the MSigDB database (https://www.gsea-msigdb.org/gsea/msigdb/index.jsp) utilizing the keyword “SNARE” (Table S1, Supplemental Digital Content 1). Transcriptomic and clinical prognostic data for the IMvigor210 immunotherapy cohort were acquired using the IMvigor210CoreBiologies R package. The expression of SRGs in the noncancerous and NSCLC cell lines were obtained from the Cancer Cell Line Encyclopedia (https://sites.broadinstitute.org/ccle/datasets) database.

### 2.2. Differential expression analysis

The TCGA-LUSC cohort comprised 501 LUSC cases and 51 normal tissue samples. Differential expression genes between LUSC and non-tumor samples were identified using the DESeq2 and limma packages in R, with thresholds set at *P* < .05 and an absolute log (fold change) > 1.

### 2.3. Model construction and evaluation

Univariate Cox regression analysis was conducted to select SRGs related to the overall survival (OS) of LUSC (*P* < .05). Next, Least Absolute Shrinkage and Selection Operator (LASSO)-Cox regression was performed using the glmnet package with 10-fold cross-validation, using deviance as the evaluation metric (type.measure = “deviance”). The optimal lambda value (lambda.min) was selected to minimize the cross-validation error. To further validate the stability of the model, we performed 1000 bootstrap resamplings and calculated the inclusion frequency of each gene. The prognostic signature was constructed using the following formula: Riskscore = Σ(cofi * mRNAi). The cofi and mRNAi represent the coefficient and expression level of gene i, respectively. In both the training and validation cohorts, patients were stratified into high-/low-risk groups based on median risk score. We employed Kaplan–Meier survival curve analysis and log-rank test to discern the disparities between these groups. To appraise the precision of the SRGs risk model in forecasting patient survival outcomes, we generated time-dependent receiver operating characteristic (ROC) curves using the timeROC R package.

### 2.4. Nomogram development and assessment

Univariate and multivariate Cox regression analyzed prognostic factors including age, gender, TNM stage, clinical stage, risk score, and smoking status. We constructed a new nomogram using variables with *P*-values < .05 from a multivariate Cox proportional hazards model to predict the prognosis of LUSC. The predictive accuracy of the model was evaluated using calibration curves and ROC curves. Additionally, we assessed and compared the clinical utility of the nomogram by incorporating clinical outcomes through 1-year decision curve analysis (DCA).

### 2.5. Enrichment analysis

Enrichment analyses were performed on differentially expressed genes between the high-risk and low-risk groups using Gene Ontology (GO) and Kyoto Encyclopedia of Genes and Genomes (KEGG) pathways. These analyses were conducted using the clusterProfiler R package.^[11]^ Statistical significance was set at a false discovery rate threshold of <0.05. The Hallmark gene sets were acquired from the MSigDB database to conduct gene set enrichment analysis. Subsequent visualization of the enrichment results was generated utilizing the GseaVis package.

### 2.6. Assessment of the immune microenvironment

The tumor immune microenvironment was assessed using the IOBR package. Infiltration levels of immune cells in the TCGA-LUSC cohort were evaluated by applying the CIBERSORT, EPIC, xCell, MCPcounter, TIMER, and quantiseq algorithms. The ESTIMATE algorithm was employed to calculate the Stromal score, Immune score, ESTIMATE score, and tumor purity. Spearman correlation analysis was performed to assess theassociations of the risk score and its constituent genes with the aforementioned immune landscape metrics, as well as with the expression of immune checkpoint genes, including CD274 molecule (*CD274*), cytotoxic T-lymphocyte associated protein 4 (*CTLA4*), hepatitis A virus cellular receptor 2 (*HAVCR2*), lymphocyte-activation gene 3 (*LAG3*), programmed cell death 1 (*PDCD1*), programmed cell death 1 ligand 2 (*PDCD1LG2*), sialic acid-binding Ig-like lectin 15 (*SIGLEC15*), and T cell immunoreceptor with Ig and ITIM domains (*TIGIT*). Differences in immune cell infiltration between the high- and low-risk groups were compared using the Wilcoxon rank-sum test. Additionally, Spearman correlation was used to evaluate the relationship between cancer-associated fibroblasts and myeloid cells.

### 2.7. Drug sensitivity assessment

We evaluated drug sensitivity with the pRRophetic package^[12]^ and compared high-/low-risk groups using Wilcoxon tests. Additionally, Pearson correlation analysis was performed to examine the relationship between the expression levels of feature genes and drug sensitivity.

### 2.8. Cell culture and transfection

NCI-H520 cells were maintained in RPMI-1640 medium supplemented with 10% fetal bovine serum (FBS) and 1% penicillin/streptomycin at 37°C under 5% CO_2_. Cells were passaged at 80% to –90% confluence using 0.25% trypsin-ethylenediaminetetraacetic acid and reseeded at a 1:3 ratio. For gene silencing, bet1 golgi vesicular membrane trafficking protein (*BET1*)-targeting siRNA and scrambled control siRNA (designed and synthesized by Guangzhou RiboBio Co., Ltd.) were transfected into cells using Lipofectamine 3000 according to the manufacturer’s protocol. Transfected cells were harvested 48 hours post-transfection for downstream analysis.

### 2.9. Western blot analysis

Transfected NCI-H520 cells were washed thrice with ice-cold phosphate-buffered saline (PBS) and lysed in radioImmunoprecipitation assay buffer (Beyotime) containing protease inhibitors. After 30-min ice incubation, lysates were sonicated (3 × 10 sec bursts) and centrifuged (13,000 rpm, 10 minutes, 4°C). Supernatants were quantified by bicinchoninic acid assay (Beyotime). 40 μg proteins/lane were separated by sodium dodecyl sulfate - polyAcrylamide gel electrophoresis and transferred to polyvinylidene difluoride membranes. Membranes were blocked with 3% BSA for 1 hour at RT, then incubated overnight at 4°C with primary antibodies: anti-BET1 (1:1000) and anti-GAPDH (1:2000). After tris-buffered saline with tween (TBST) washing (3 × 10 minutes), membranes were probed with HRP-conjugated secondary antibodies (1:5000) for 1 hour at RT. Protein bands were visualized using ECL after final TBST washes.

### 2.10. RNA extraction and quantitative qRT-PCR

Total RNA extraction occurred 48 hours following transfection via the Trizol technique (Invitrogen, USA), which was followed by cDNA synthesis employing RT-PCR with SYBR Green reagents (Thermo Fisher Scientific, USA). Amplification was performed using the PCR-7500 Real-Time PCR System (Applied Biosystems, USA), and internal controls were normalized to GAPDH. Fold change quantifications were computed using the 2^-ΔΔCq approach. Primer details can be found in Table S2, Supplemental Digital Content 2.

### 2.11. Colony formation assay

In each well of 6-well plates, 200 cells were cultivated in 2 ml of 1640 medium enriched with 10% FBS under typical settings for about 2 weeks to facilitate colony development. Subsequently, Cells were fixed with 4% paraformaldehyde (15 minutes), washed with PBS (3×), and stained with 0.1% crystal violet (5 minutes). Colonies were tallied and scrutinized.

### 2.12. Transwell invasion assay

Transwell invasion assays were performed using 24-well plates with 8 μm pore inserts. The lower chamber contained 600 μl RPMI-1640 supplemented with 10% FBS, and the upper chamber was seeded with 8000 cells in 200 μl serum-free medium. After 24 hours incubation, nonmigrated cells on the upper membrane surface were removed by cotton swab. Membranes were washed with PBS, fixed with 4% paraformaldehyde, and stained with 0.1% crystal violet. Invasive cells were quantified by counting cells in 5 random fields per membrane under a microscope.

### 2.13. Wound healing assay

Upon transfection, cells were seeded at a density of 3 × 10^5 cells per well in 6-well plates and grown until they reached approximately 90% confluence. Using a 100 ul pipette tip, we created a scratch across the middle of each well. At 0 and 24 hours, we took pictures at 3 distinct points along each scratch to document wound dimensions and evaluate recovery rates among groups.

### 2.14. Statistical analysis

Bioinformatic analyses were performed using R software. The differences between the 2 groups were evaluated using the Mann–Whitney U test, with a *P*-value <.05 indicating statistical significance.

## 3. Results

### 3.1. Construction of SRG-derived prognostic signature

**Figure 1.**
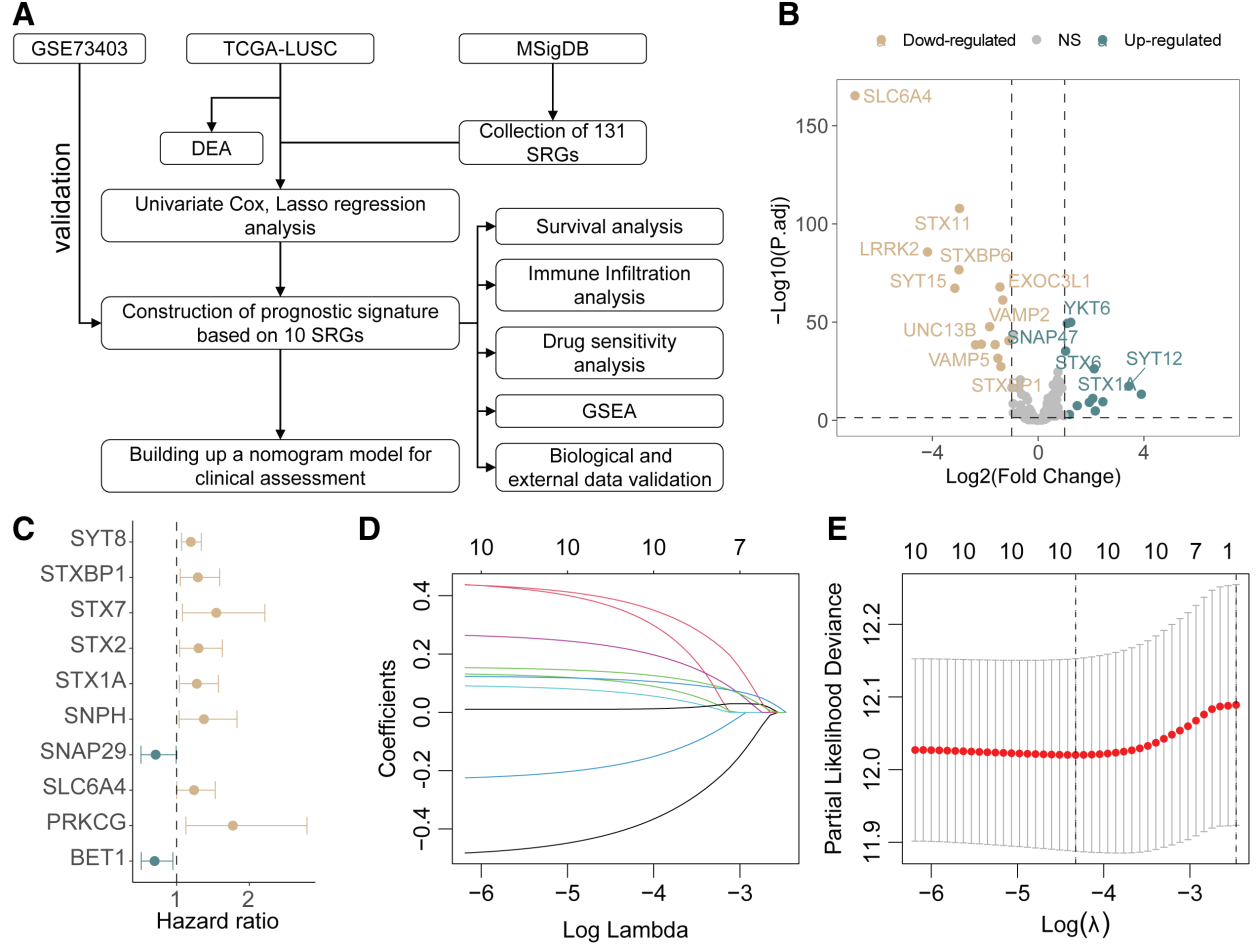
Prognostic feature derived from SRGs based on LASSO for LUSC. (A) The workflow of the study. (B) Volcano plot for differential expression analysis of SRGs based on the TCGA-LUSC cohort. (C) SRGs related to prognosis obtained from univariate Cox analysis. (D) LASSO coefficient profile plots of prognostic-related SRGs. (E) Penalty plot for the LASSO regression analysis.

Our study workflow is illustrated in Figure 1A. Twenty-six SRGs displayed abnormal expression levels in LUSC, with 12 being notably elevated and 14 being noticeably reduced (Fig. 1B). Ten SRGs were identified as having significant associations with LUSC patient OS (*P* < .05). Among these, synaptosome-associated protein 29 (*SNAP29*) and *BET1* acted as favorable prognostic factors, while the remaining 8 genes, including synaptotagmin 8 (*SYT8*), syntaxin binding protein 1 (*STXBP1*), syntaxin 7 (*STX7*), syntaxin 2 (*STX2*), syntaxin 1A (*STX1A*), syntaphilin (*SNPH*), solute carrier family 6 member 4 (*SLC6A4*), and protein kinase C gamma (*PRKCG*), were identified as unfavorable prognostic indicators for LUSC (Fig. 1C, Supplementary Materials: Figure S1, Supplemental Digital Content 3). Based on these ten prognosis-related SRGs, we employed LASSO regression analysis to construct a risk signature specific to LUSC (Fig. 1D, E). The signature was calculated using the following formula: RiskScore = 0.111 × *SYT8* + 0.135 × *STXBP1* + 0.378 × *STX7* + 0.011 × *STX2* + 0.218 × *STX1A* + 0.067 × *SNPH* − 0.174 × *SNAP29* + 0.103 × *SLC6A4* + 0.346 × *PRKCG* − 0.402 × *BET1*. This signature represents a weighted sum of the expression levels of the aforementioned genes, with positive coefficients indicating genes associated with poor prognosis and negative coefficients suggesting genes linked to better outcomes in LUSC patients.

### 3.2. Performance evaluation of the prognostic signature derived from SRGs

**Figure 2.**
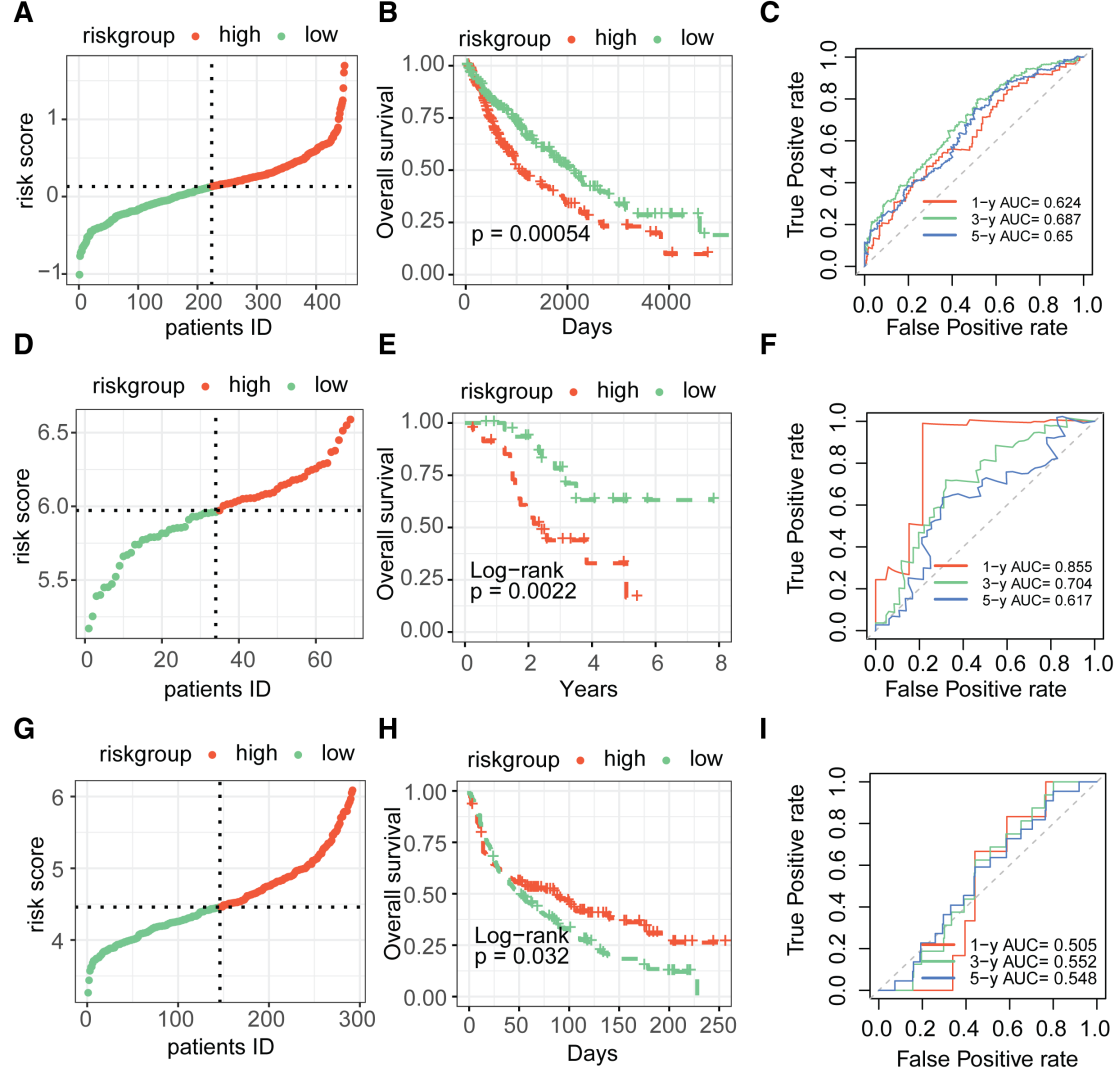
Performance evaluation of the risk feature derived from SRGs for LUSC. (A) The risk score distribution of LUSC patients, (B) the KM survival curves and (C) the ROC curves in the TCGA-LUSC cohort. (D) The risk score distribution of LUSC patients, (E) the KM survival curves, and (F) the ROC curves in the GSE73403 cohort. (G) The risk score distribution of LUSC patients, (H) the KM survival curves, and (I) the ROC curves in the GSE30219 cohort.

Based on the formulation described above, risk scores were calculated for the TCGA-LUSC, GSE73403, and GSE30219 cohorts. Subsequent analysis revealed that in the TCGA-LUSC cohort, the high-risk group had a significantly poorer prognosis compared to the low-risk group (Fig. 2A, B). The area under the curve (AUC) values of the risk score for predicting 1‑, 3‑, and 5‑year OS were 0.624, 0.687, and 0.65, respectively (Fig. 2C). Similarly, in the GSE73403 cohort, the high-risk group showed significantly worse survival outcomes than the low-risk group (Fig. 2D, E), with corresponding AUC values of 0.855, 0.704, and 0.617 for predicting 1‑, 3‑, and 5‑year OS, respectively (Fig. 2F). Consistent with these findings, the high-risk group in the GSE30219 cohort was also associated with significantly reduced survival (Fig. 2G, H), and the AUC values for the risk score in this cohort were 0.505, 0.552, and 0.548 at 1, 3, and 5 years, respectively (Fig. 2I). Collectively, these results indicate that the SRG-derived risk score can effectively stratify LUSC patients into distinct high- and low-risk subgroups.

### 3.3. Construction of a nomogram incorporating smoking status and the SRGs-derived risk signature

**Figure 3.**
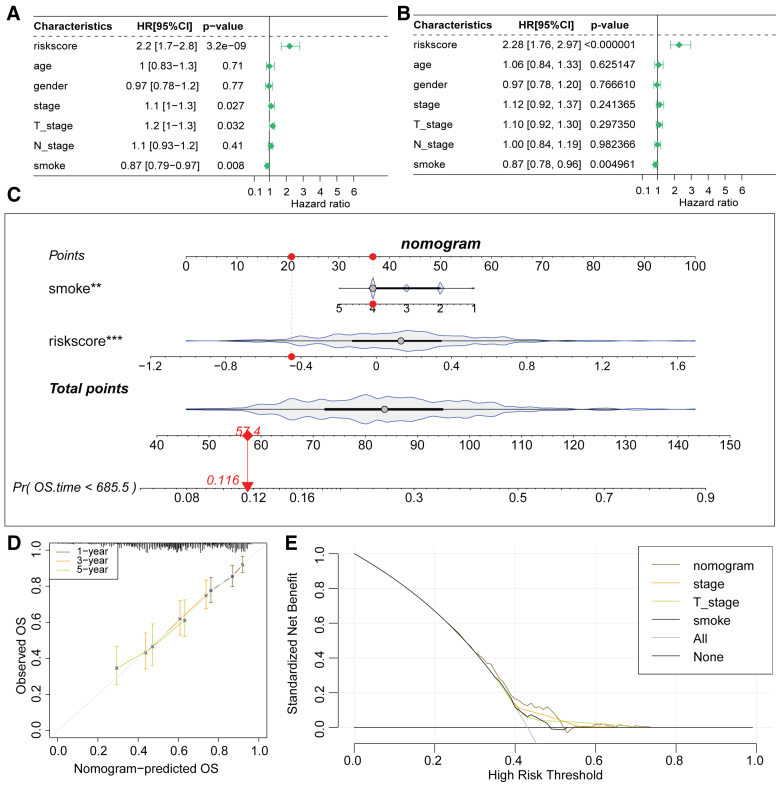
Development and evaluation of the nomogram model based on SRGs. (A) Univariate Cox analysis and (B) multivariate Cox analysis showing the correlation of overall survival with risk score and clinicopathological factors. (C) Nomogram model constructed by smoke and risk score. (D) Calibration curves for 1 year, 3 years, and 5 years were used to assess the calibration and accuracy of the nomogram model. (E) Decision curve analysis evaluates the performance advantage of the nomogram over other prognostic factors.

To determine the clinical independence of our model as a prognostic indicator for LUSC patients, we conducted univariate and multivariate Cox regression analyses. Our findings found that the risk signature and smoking history were the sole independent prognostic factors capable of predicting survival rates in LUSC patients (Fig. 3A, B). Therefore, we developed a novel nomogram that integrates the independent factors of smoking status and the risk score to estimate 1-, 3-, and 5-year OS probabilities for LUSC patients (Fig. 3C). Calibration curve analysis confirmed that this nomogram possesses robust predictive accuracy (Fig. 3D). Furthermore, DCA demonstrated that our nomogram outperformed other individual factors in predicting patient outcomes, thereby underscoring its significant potential for practical application in clinical settings (Fig. 3E).

### 3.4. Association of SRGs and their derived risk signature with the tumor immune microenvironment

**Figure 4.**
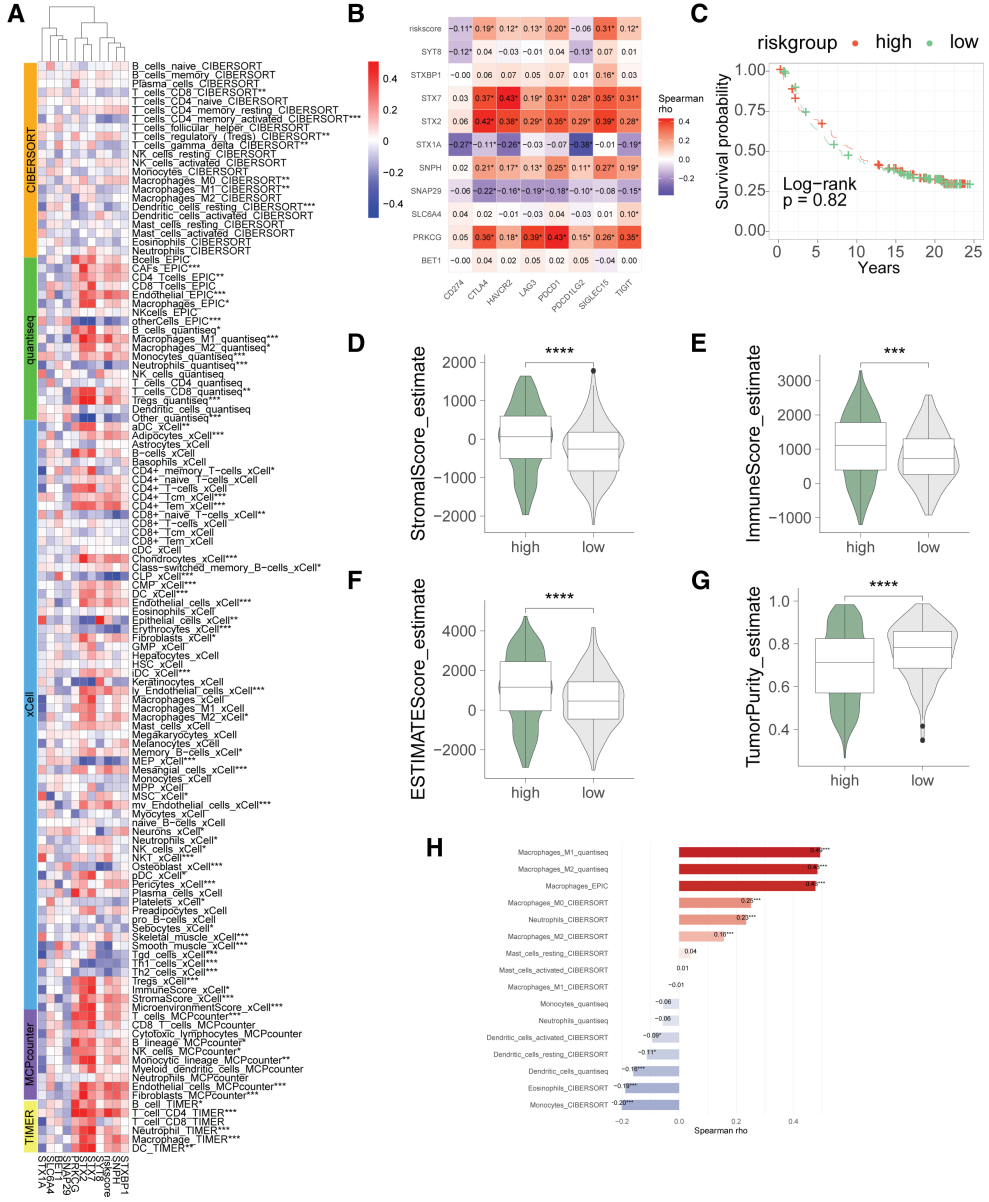
Relationship between SRGs and their derived features with the tumor immune microenvironment in LUSC. (A) Heatmap depicting the correlations between the risk score, the expression of its constituent SRGs, and immune cell infiltration levels. Asterisks (*) indicate statistically significant differences in immune cell infiltration between the high- and low-risk groups. (B) Heatmap showing correlations between the risk score, the expression of constituent SRGs, and immune checkpoint gene expression. (C) Kaplan–Meier survival curves comparing high- and low-risk groups in the IMvigor210 cohort. (D-G) Differences in ESTIMATE algorithm scores between the high- and low-risk groups. (H) Correlations between cancer-associated fibroblast (CAF) infiltration and infiltration of myeloid cell populations. *P < .05, **P < .01, ***P < .001, ****P < .0001.

Subsequently, we examined the correlations between the risk score, the expression levels of its constituent genes, and immune cell infiltration. The results showed that the risk score was significantly associated with the infiltration of various immune cells, including T cells, B cells, macrophages, cancer-associated fibroblasts (CAFs), and dendritic cells. Notably, multiple algorithms consistently revealed a significant positive correlation with regulatory T cells (Tregs) and CD4^+^T cells, and a significant negative correlation with CD8^+^T cells, Th1, and Th2 cell subsets. Moreover, the abundances of these immune cell populations differed markedly between the high- and low-risk groups (Fig. 4A). In addition, we found that the risk score was significantly positively correlated with the expression of multiple immune checkpoint genes, including *CTLA4*, *HAVCR2*, *LAG3*, *PDCD1*, *SIGLEC15*, and *TIGIT*, with similar correlation trends observed for *STX7*, *STX2*, *SNPH*, and *PRKCG* (Fig. 4B). However, no significant survival differences between the high- and low-risk groups were observed in the immunotherapy-treated cohort (Fig. 4C). In addition, we observed that the high-risk LUSC patients had higher stromal and immune scores, as well as lower tumor purity when compared to the low-risk patients (Fig. 4D–G). Furthermore, CAF infiltration levels were strongly associated with the infiltration of various myeloid cell subsets, including macrophages, neutrophils, dendritic cells, eosinophils, and monocytes (Fig. 4H). These findings suggest that SRGs, as well as their derived signature are associated with the tumor immune landscape.

### 3.5. Association of SRGs and their derived features with drug sensitivity

**Figure 5.**
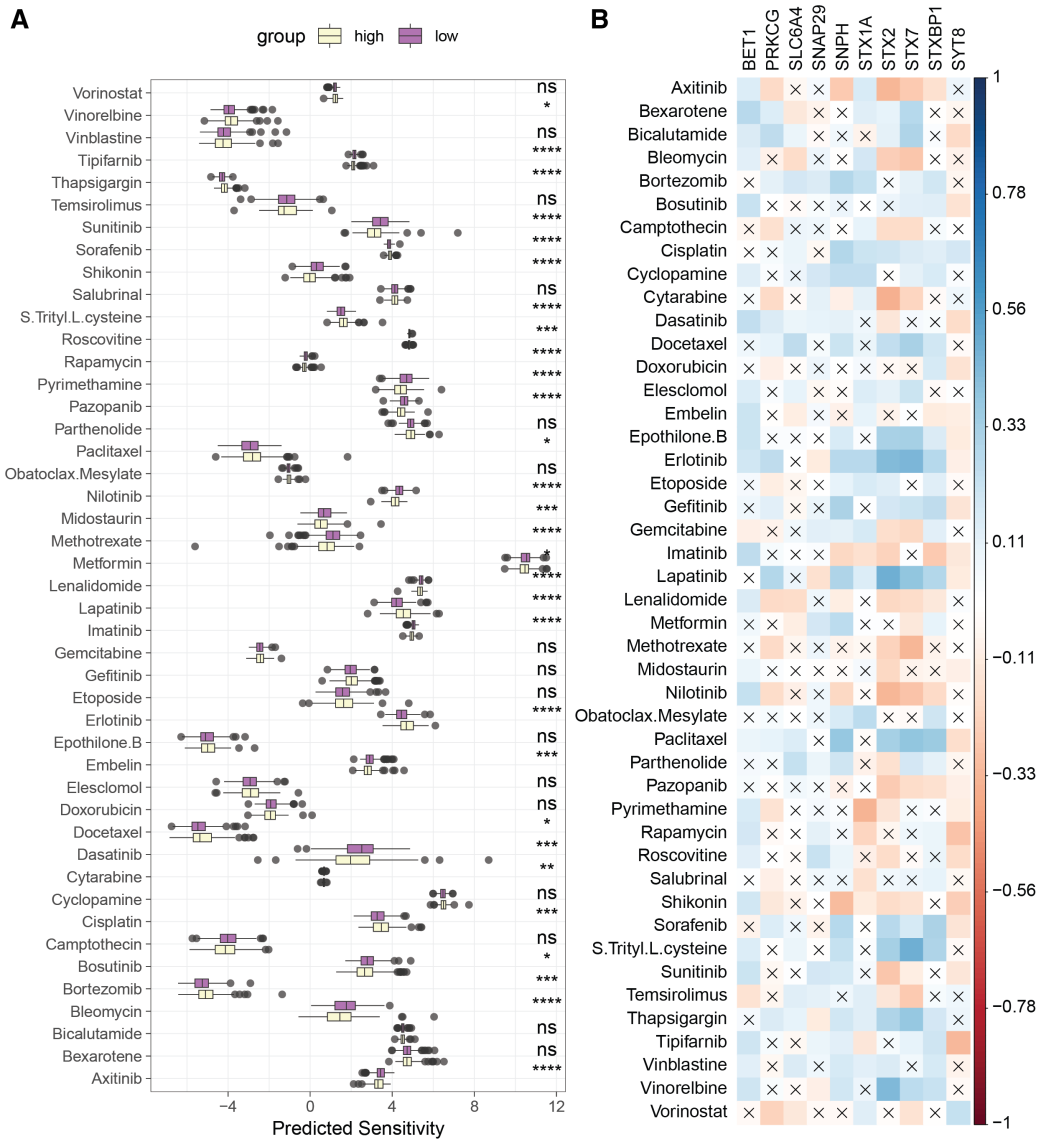
Relationship between SRGs and their derived features with drug sensitivity. (A) Comparison of sensitivity differences to 45 drugs between high-risk and low-risk LUSC patients. (B) Heatmap showing the correlation between the 10 SRGs constituting the risk feature and sensitivity to 45 drugs. ns, not significant, * *P* < .05, ** *P* < .01, *** *P* < .001, **** *P* < .0001.

We further calculated the sensitivity of the TCGA-LUSC cohort to 45 drugs and compared differences between the risk groups. The results showed that compared to the high-risk group, patients in the low-risk group had higher sensitivity to 10 drugs but lower sensitivity to 19 drugs (Fig. 5A). It was revealed that the SRGs constituting the risk signature had complex correlations with drug sensitivity, where *BET1* was positively correlated with the sensitivity to most drugs (Fig. 5B). These findings suggest that SRGs and the signature are associated with chemotherapeutic response.

### 3.6. Clinical pathological characteristics and molecular mechanisms associated with the SRG-derived risk signature

**Figure 6.**
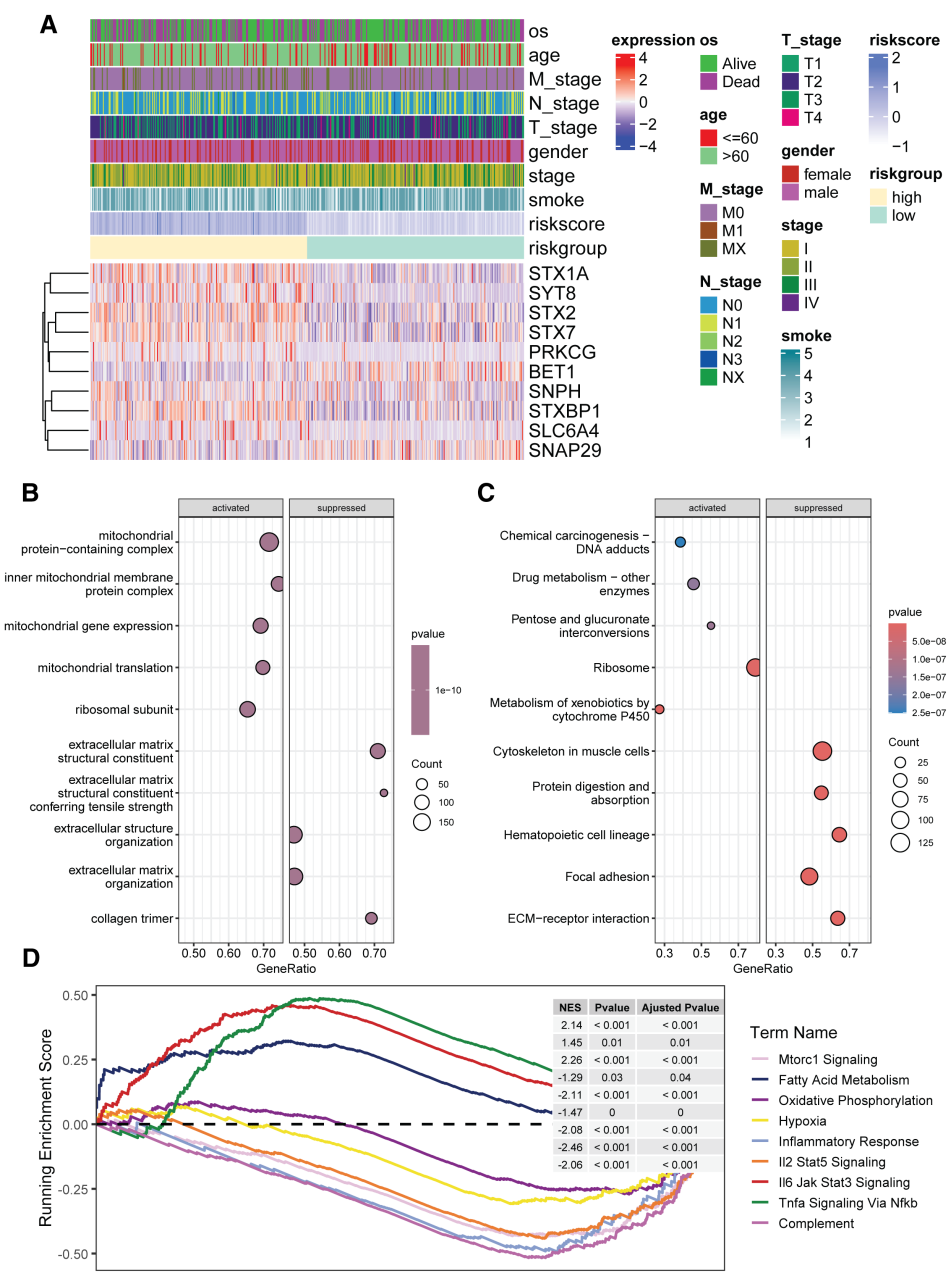
Association of the risk feature derived from SRGs with clinicopathological characteristics and gene expression. (A) Heatmap of the expression of SRGs constituting the risk feature and annotations of clinicopathological characteristics. (B) Gene Ontology (GO) enrichment analysis results. (C) Kyoto Encyclopedia of Genes and Genomes (KEGG) pathway enrichment analysis results. (D) Hallmark gene set enrichment analysis results.

Figure 6A presents the heatmap of expression profiles for SRGs in the signature, annotated with clinical pathological features. Consequently, *BET1* and *SNAP29* were found to be highly expressed in low-risk LUSC cases, while the other SRGs exhibited lower expression levels. When comparing risk scores across various clinical and pathological subgroups, it was observed that patients who were alive had lower risk scores than those who had deceased. Interestingly, T1-stage patients showed higher risk scores than T2-stage, while 1-year smokers had higher scores than 4-year smokers (Supplementary materials: Figure S2, Supplemental Digital Content 4). Furthermore, our results showed that genes involved in mitochondrial processes were markedly upregulated in the high-risk group, while genes associated with extracellular matrix (ECM)-related components and functions were markedly downregulated. Within the KEGG gene set, we noted that genes related to ribosome and drug metabolism pathways were significantly activated in the high-risk group, whereas pathways involving focal adhesion, ECM-receptor interaction, and protein digestion and absorption were significantly inhibited in this group (Fig. 6B, C). Furthermore, we observed dysregulated expression of Hallmark gene sets, including MTORC1 signaling, fatty acid metabolism, oxidative phosphorylation, hypoxia, inflammatory response, IL2 STAT5 signaling, IL6 JAK STAT3 signaling, TNFA signaling via NFkB, and complement, across risk score subgroups (Fig. 6D), indicating an association between the SRG signature and both metabolic reprogramming and immune evasion.

### 3.7. Screening of key SRG and its biological functions

**Figure 7.**
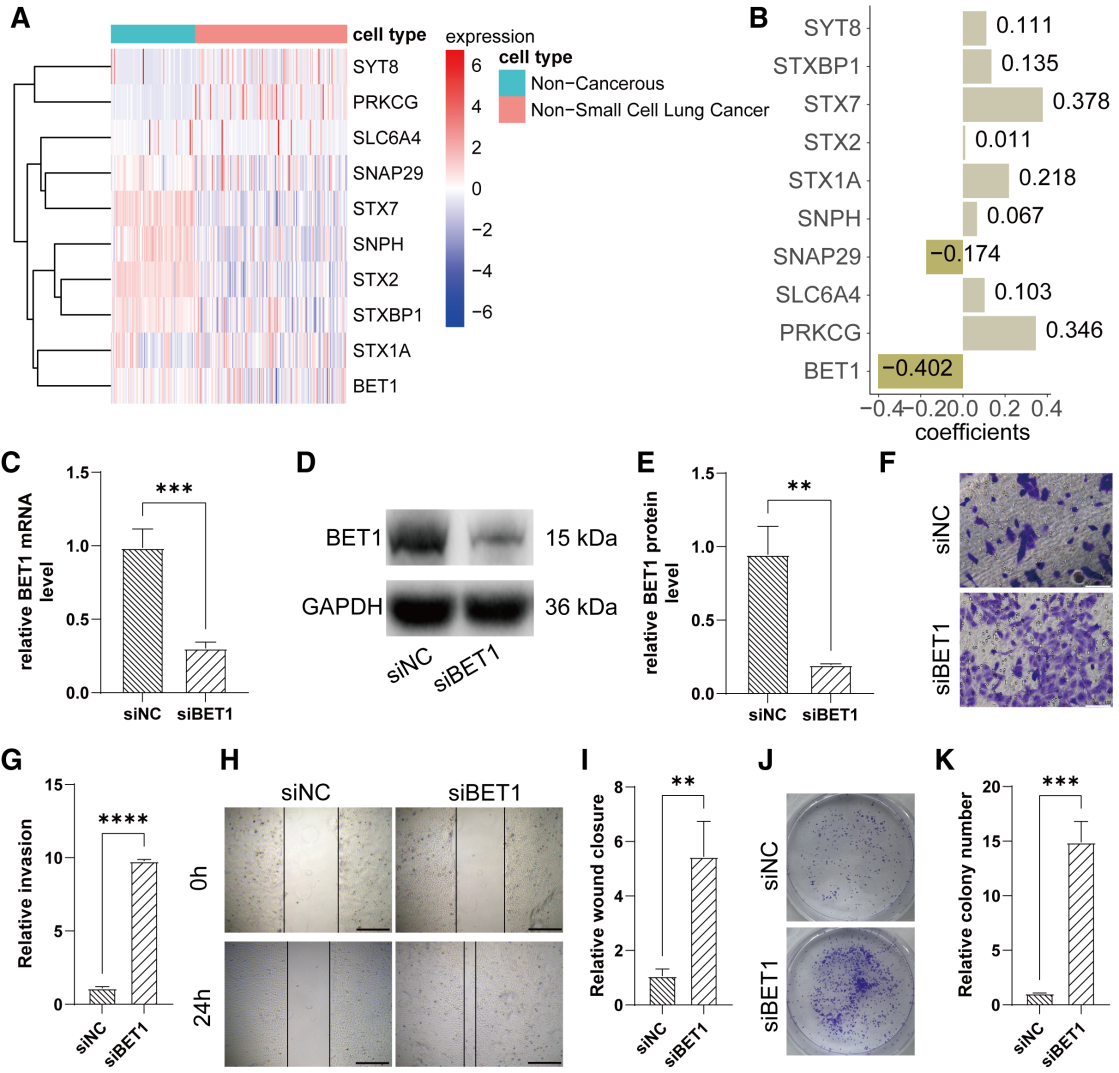
Screening of key SRGs and verification of their biological function. (A) Distribution of coefficients of SRGs constituting the risk feature. (A) The heatmap of gene expression of BET1 in noncancerous cells and NSCLC. (B) The coeffieients for each gene in the signature. (C) Comparison of the BET1 gene expression between the siNC and siBET1 cells. (D) Western blot images of the BET1 protein detected by Western blot assay. (E) Comparison of the BET1 protein expression between the siNC and siBET1 cells. (F) Representative images of crystal violet-stained invaded cells from each group. (G) Comparison of the relative invasion in each group. (H) Representative bright field images of the wound healing assay from each group. (I) Comparison of the relative wound closure in each group. (J) Representative crystal violet-stained images of colonies from each group. (K) Comparison of the relative colony number in each group. Bar charts represent mean ± SD.**P < .01, and ***P < .001. Scale bars, 100 μm (F) and 500 μm (H).

First, we compared the expression of SRGs between NSCLC cell lines and noncancerous cell lines, and found significant abnormal expression. There is also heterogeneity among different NSCLC cell lines (Fig. 7A). Among SRGs comprising the risk signature, *BET1* was the most impactful contributor based on its highest absolute coefficient value in our risk model (Fig. 7B), prompting further investigation into its functional role in LUSC. Knockdown of *BET1* via siRNA in NCI-H520 cells (with transfection efficiency confirmed by western blot and RT-PCR; Fig. 7C–E) elicited pronounced pro-tumorigenic effects. Transwell invasion assays demonstrated a signifcant increase in invasive capacity (*P* < .0001) in siBET1-treated cells compared to siNC controls (Fig. 7F,G). Complementary functional assays revealed that BET1 suppression significantly enhanced wound closure in scratch assays (Fig. 7H,I; *P* < .01) and increased colony-forming capacity (Fig. 7J,K; *P* < .001). These collective findings establish *BET1* as a critical suppressor of proliferative, migratory, and invasive phenotypes in LUSC cells.

## 4. Discussion

LUSC remains one of the most lethal malignancies globally.^[13]^ Despite recent advancements in diagnostics and therapies, the overall prognosis for patients with LUSC remains unfavorable, partly owing to the absence of effective prognostic markers. Recent immunotherapies have shown promise in improving survival and quality of life for LUSC patients; however, owing to the variability and complexity of the tumor immune microenvironment, only a small subset of patients benefit from such treatments.^[14–17]^ Consequently, there is an urgent need to develop prognostic risk models and nomograms to guide clinical treatment strategies for LUSC, aiming to ultimately enhance patient outcomes.

SRGs influence tumor progression, immune responses, and therapy efficacy in multiple cancers. This supports constructing SRG-based prognostic risk models.^[18]^ In this study, we curated 131 SRGs from the MSigDB database and identified 10 SRGs associated with LUSC prognosis. We then developed a risk signature comprising these 10 SRGs. Leveraging machine learning techniques, such as LASSO regression, random survival forests, and support vector machines, enables efficient feature selection from high-dimensional transcriptomic data, identification of robust prognostic biomarkers, and construction of predictive models with optimized generalization performance. In cancer biomarker discovery, machine learning not only improves the sensitivity and specificity of candidate gene identification but also facilitates the integration of multi-omics data (e.g., genomics, transcriptomics, proteomics) to uncover hidden patterns and interactions that are difficult to detect using conventional statistical methods.^[19]^ Our findings demonstrate that the risk model, based on SRG expression profiles, serves as an independent prognostic predictor for LUSC. Additionally, this signature can be combined with the clinical features of LUSC patients to create a nomogram. These discoveries suggest that risk models based on SRG expression profiles hold potential clinical application value in guiding therapeutic decisions and improving patient prognosis.

Among the 10 SRGs identified, multiple studies highlight their roles in cancer progression and therapy resistance. SNAP29, a key regulator of autophagosome-lysosome fusion, has been implicated in modulating cisplatin sensitivity in ovarian cancer through O-GlcNAcylation, which disrupts the syntaxin 17 (*STX17*)–*SNAP29*–vesicle associated membrane protein 8 (*VAMP8*) complex and inhibits autophagic flux.^[20–22]^ *SYT8* facilitates metastasis in pancreatic and gastric cancers by activating the troponin I2, fast skeletal type (*TNNI2*)/*ERR*α/sirtuin 1 (*SIRT1*) axis and confers resistance to fluorouracil.^[23,24]^ It also forms a read-through fusion transcript SYT8/*TNNI2* in bladder cancer, underscoring its tumor-specific alternative splicing.^[25]^ *STXBP1* is associated with adverse outcomes in lung adenocarcinoma, where its membrane localization correlates with poor prognosis and may influence exocytic processes.^[26,27]^ *STX7* exhibits context-dependent roles: it promotes invadopodia formation and invasion in breast cancer through coordination with vesicle associated membrane protein 2/3 and *STX4*, yet shows reduced expression in advanced melanoma, suggesting a stage-specific function.^[28,29]^ *STX2* promotes colorectal cancer growth by increasing exosome secretion or upregulating exosome component 4 (*EXOSC4*).^[10,30]^ Moreover, it engages in a positive feedback loop with TNF receptor associated factor 6 (*TRAF6*) to activate NF-κB signaling, thereby accelerating metastasis.^[31]^ *STX1A* is upregulated in bladder cancer and contributes to disease progression,^[32]^. Notably, a 3-gene signature including *STX1A*, hypoxia inducible factor 1 subunit alpha (*HIF1A*), and C-C motif chemokine receptor 7 (*CCR7*) effectively stratifies early-stage NSCLC patients independent of TNM stage.^[33]^ Recent studies also reveal its role in mitochondrial regulation and ferroptosis modulation in gastric cancer, impacting chemoresistance.^[34]^ *SNPH*, originally identified as a mitochondrial docking protein, inhibits tumor cell motility and serves as a biphasic biomarker in prostate cancer, with reduced expression in metastatic disease.^[35,36]^ It also regulates neutrophil migration within the tumor microenvironment, influencing immunosuppressive cell dynamics.^[37]^ *SLC6A4*, beyond its classical role in serotonin transport, is linked to poor survival in colorectal cancer and correlates with cancer-related fatigue and analgesic morphine dosage in lung cancer patients.^[38–40]^ Aberrant expression of *SLC6A4* in cancer impacts cell proliferation and apoptosis, correlating with tumorigenesis and progression.^[40]^ *PRKCG*, a protein kinase C gamma isoform, contributes to oncogenic signaling and hepatocellular carcinoma susceptibility via nonsynonymous SNPs that affect protein structure and function.^[41]^ Its noncoding variants may also disrupt epigenetic and miRNA-mediated regulatory networks.^[42]^ Finally, *BET1* (also termed *MDRP*) has been associated with chemotherapy response in lung cancer,^[43]^ was functionally validated as a tumor suppressor in lung cancer through our experimental studies. Collectively, these SRGs influence diverse oncogenic processes, including vesicular trafficking, metabolic reprogramming, immune modulation, and drug resistance, highlighting their utility as multifunctional biomarkers and potential therapeutic targets in precision oncology.

Tumor recurrence and survival are closely linked to antitumor immunity mediated by immune cell infiltration, particularly CD8 + T cells or neutrophils into the tumor microenvironment.^[44,45]^ In our study, the SRG-derived risk score was significantly associated with distinct immune infiltration patterns: high-risk patients exhibited lower CD8^+^ T cell infiltration but higher levels of Tregs and CAFs, suggesting a more immunosuppressive tumor milieu. Notably, *STXBP1* correlated with CD8 + T cell infiltration, and its interaction with *STX1* drives up to 50% of cytotoxic activity in NK/CD8 + T cells.^[46]^ Conversely, most risk-associated SRGs showed an inverse correlation with M1 macrophage infiltration, indicating that elevated SRG expression might blunt pro-inflammatory, antitumor immune responses.^[47]^ Furthermore, risk score was positively correlated with multiple immune checkpoint genes (e.g., *CTLA4*, *LAG3*, *PDCD1*, *TIGIT*), aligning with the observed enrichment of immunosuppressive cell populations. While enhancing recruitment of CD4^+^ T lymphocytes can potentiate immunotherapy efficacy,^[48]^ our findings reveal that SRGs were negatively correlated with CD4^+^ T cell infiltration, suggesting potential hurdles for effective immune activation in high-risk patients. Collectively, these results indicate that SRGs not only stratify patients by prognosis but also delineate distinct immunological landscapes, where high-risk tumors are characterized by suppressed cytotoxic immune infiltration, elevated inhibitory signaling, and enriched stromal elements, which may underlie reduced responsiveness to immune checkpoint blockade. Targeting unfavorable SRGs or modulating their downstream pathways could thus represent a promising approach to remodel the tumor immune microenvironment, potentially converting immunologically “cold” tumors into “hot” ones that are more responsive to immunotherapy.

A critical question that arises from this association is the relationship between our SRG-signature and response to immune checkpoint blockade (ICB). Interestingly, when applied to the IMvigor210 cohort (a cohort of metastatic urothelial carcinoma patients treated with anti-PD-L1 atezolizumab), our risk signature did not show a significant association with OS. This null result is informative and suggests that the prognostic value of SRGs may be cancer-type specific and/or that the immune evasion mechanisms driven by SRGs are not solely dependent on the PD-1/PD-L1 axis. It is possible that the immunosuppressive microenvironment characterized by high SRG-risk scores (e.g., rich in Tregs and M2 macrophages, deficient in CD8 + T cells) may be primarily mediated by other mechanisms, making it less susceptible to PD-1/PD-L1 monotherapy. Rather than diminishing their therapeutic relevance, this insight redirects the potential of targeting SRGs. The significant biological role of BET1, validated in our experiments, underscores that SRGs are functional drivers of tumor aggressiveness. Therefore, targeting specific SRGs (e.g., using siRNA, small molecule inhibitors, or antibodies) could directly inhibit tumor invasion and metastasis.^[31]^ Furthermore, given their role in shaping a ‘cold’ tumor immune microenvironment, SRG inhibition could be explored as a novel combination strategy to sensitize tumors to immunotherapy. For instance, inhibiting an unfavorable SRG (e.g., *STXBP1*) might inhibit macrophage infiltration and reverse immunosuppression, potentially converting a non-responder into a responder when combined with ICB.^[49]^ This combination approach warrants urgent investigation in preclinical models of LUSC.

These collective findings establish that our SRG-derived signature is not only a prognostic indicator but also a reflector of a distinct and immunosuppressive tumor microenvironment in LUSC. However, this finding must be considered in the context of the inherent molecular heterogeneity of lung squamous cell carcinoma. The inherent heterogeneity of LUSC, encompassing basal, classical, secretory, and primitive molecular subtypes with distinct oncogenic drivers and immune microenvironments, presents both a challenge and an opportunity for prognostic modeling.^[50]^ While our current study stratified patients based on a pan-LUSC SRG signature, the potential interaction between SNARE expression and these intrinsic subtypes warrants further investigation. Future studies with larger cohorts should explore whether the prognostic power of our signature is enriched within specific LUSC subtypes, which could refine its clinical applicability. Furthermore, the integration of SNARE expression profiles with established mutational signatures could provide deeper insights into the mechanistic links between vesicular trafficking, carcinogenesis, and tumor evolution.^[51]^

Vesicular trafficking is a critical mechanism within cells responsible for transmembrane transport and inter-organelle communication, playing roles in drug uptake and distribution, metabolism, excretion, and resistance.^[52,53]^ As key mediators of membrane fusion, SRGs displayed complex relationships with drug sensitivity in our analysis. It has been shown that *SYT8* expression was related to fluorouracil resistance.^[24]^ Downregulation of O-GlcNAc and O-GlcNAc transferase through *SNAP29* enhances cisplatin-induced autophagy, leading to cisplatin resistance in ovarian cancer.^[54]^ Therefore, more experiments are required to elucidate the relationship between SRGs and drug sensitivity to develop targeted strategies to counteract chemoresistance.

While our SRG-based prognostic model demonstrates robust predictive power in retrospective cohorts, its clinical translation requires several key steps. First, standardization of detection methods is essential. The gene expression signature could be implemented as a clinically feasible assay (e.g., RT-qPCR or NanoString nCounter) using RNA from formalin-fixed paraffin-embedded biopsy tissues to ensure compatibility with routine workflows. Second, formal cost-effectiveness analyses are needed, since the clinical value of the signature depends on its ability to identify patients who may derive differential benefit from costly therapies such as immunotherapy or targeted agents. Such stratification could help avoid ineffective treatments, reducing both costs and toxicities; thus, future prospective studies should incorporate health-economic evaluations. Third, the added value of the SRG signature over established prognostic indicators (e.g., TNM stage, PD-L1 expression, tumor mutational burden) must be rigorously tested. Our multivariate Cox analysis already suggests prognostic independence from stage, but head-to-head comparisons in large, multi-center cohorts are required to confirm its unique contribution, potentially within integrated nomograms combining clinical, pathological, and molecular variables.

Despite the novel insights and achievements of our study, there are limitations. Firstly, the prognostic model was built using retrospective cohorts, and validation based on large-scale prospective cohorts was required. Secondly, although we assessed the biological function of *BET1*, data regarding its impact on drug sensitivity was not obtained. Moreover, the biological functions of other SRGs in LUSC remain unexplored. Finally, the lack of association in the IMvigor210 cohort highlights the need to validate the model’s predictive utility in a dedicated LUSC cohort treated with ICB. In future work, integrating multi-omics technologies, including genomics, transcriptomics, proteomics, epigenomics, and metabolomics, will be essential for a comprehensive understanding of SRG-driven tumor biology.^[55]^

## 5. Conclusion

In conclusion, we constructed an SRG-based prognostic signature that effectively assesses survival outcomes, tumor microenvironment characteristics, and therapeutic vulnerability in LUSC. Integrated with smoking history, this signature was translated into a clinically applicable nomogram. While its direct predictive value for ICB response requires further validation in LUSC cohorts, its strong association with a suppressive immune landscape and our functional validation highlight SRGs as promising therapeutic targets. Our findings highlight SRGs as promising targets requiring systematic investigation for therapeutic development.

## Author contributions

**Conceptualization:** Qingqing Chen, Jian Wang, Xiaoting Wu.

**Data curation:** Qingqing Chen, Jian Wang, Xiaoting Wu.

**Formal analysis:** Qingqing Chen, Jian Wang, Xiaoting Wu.

**Funding acquisition:** Qingqing Chen, Jian Wang, Xiaoting Wu.

**Investigation:** Qingqing Chen, Jian Wang, Xiaoting Wu.

**Methodology:** Qingqing Chen, Jian Wang, Xiaoting Wu.

**Project administration:** Qingqing Chen, Jian Wang, Xiaoting Wu.

**Resources:** Qingqing Chen, Jian Wang, Xiaoting Wu.

**Software:** Qingqing Chen, Jian Wang, Xiaoting Wu.

**Supervision:** Qingqing Chen, Jian Wang, Xiaoting Wu.

**Validation:** Qingqing Chen, Jian Wang, Xiaoting Wu.

**Visualization:** Qingqing Chen, Jian Wang, Xiaoting Wu.

**Writing – original draft:** Qingqing Chen, Jian Wang, Xiaoting Wu.

**Writing – review & editing:** Qingqing Chen, Jian Wang, Xiaoting Wu.








