# Supplementary figures and images for: Developing and validating a SNARE-based prognostic model to forecast outcomes and immune microenvironment in lung squamous cell carcinoma
Source: Medicine (Baltimore). 2026 Jul 3;105(27):e49394. doi: 10.1097/MD.0000000000049394 (PMC13337067; doi:10.1097/MD.0000000000049394)

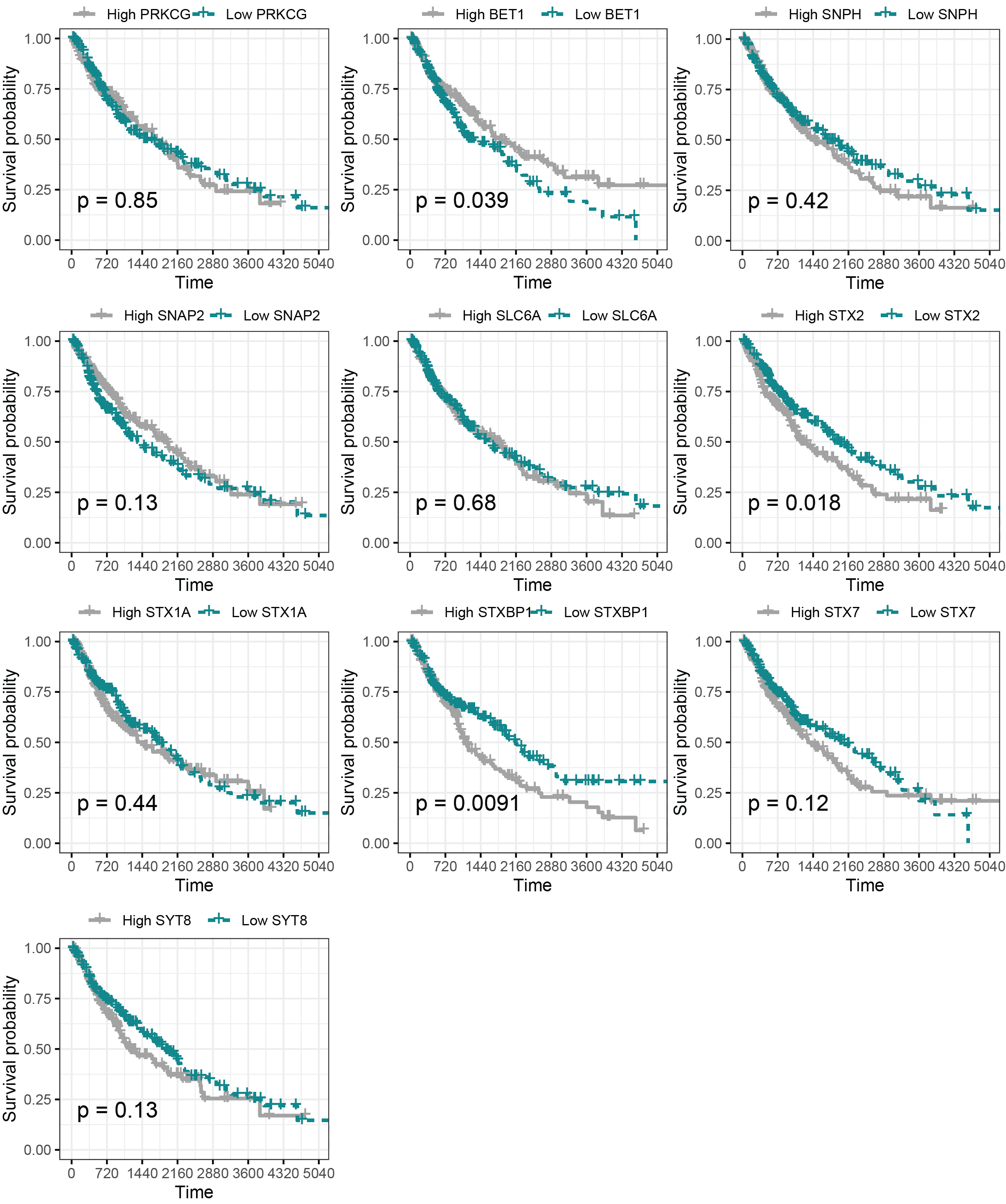

Supplement: Supplementary file 3 [file medi-105-e49394-s003.tif]

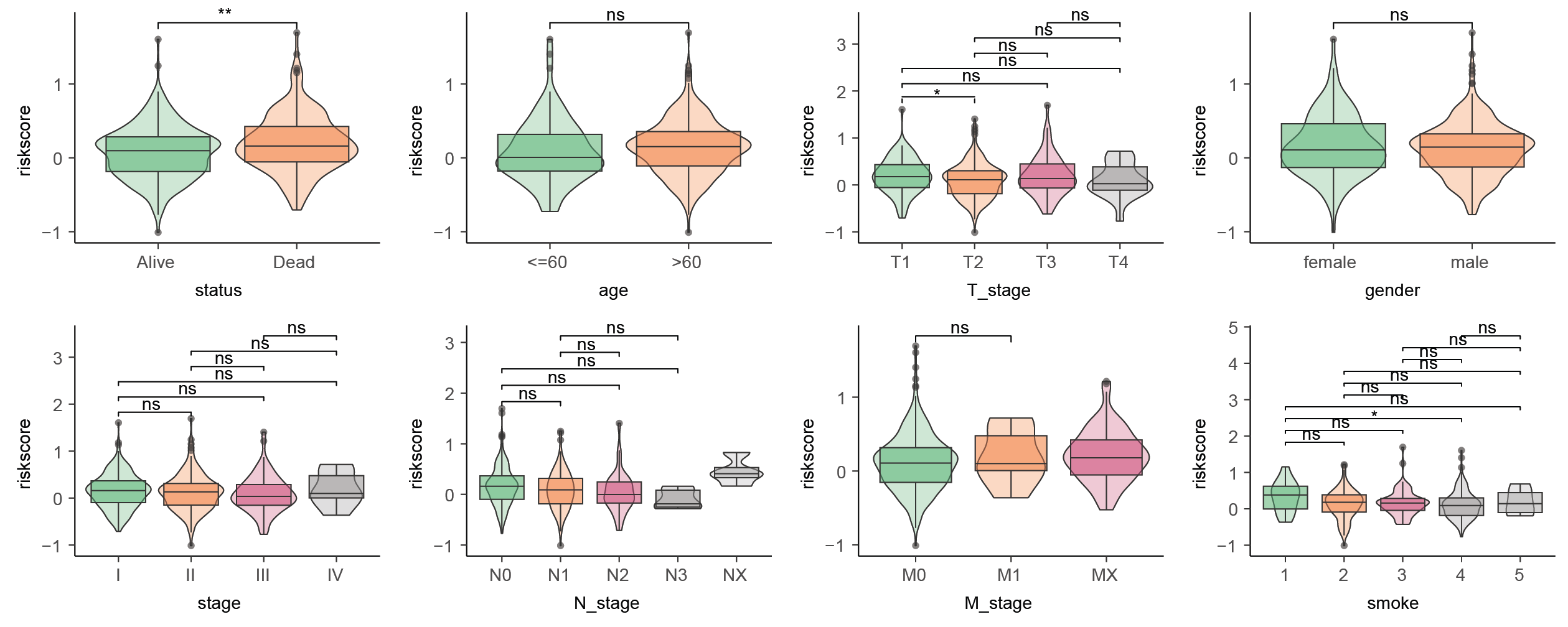

Supplement: Supplementary file 4 [file medi-105-e49394-s004.tif]
